# A novel scalable manufacturing process for the production of hydrogel-forming microneedle arrays

**DOI:** 10.1016/j.ijpharm.2015.08.049

**Published:** 2015-10-15

**Authors:** Rebecca E.M. Lutton, Eneko Larrañeta, Mary-Carmel Kearney, Peter Boyd, A.David Woolfson, Ryan F. Donnelly

**Affiliations:** School of Pharmacy, Queens University Belfast, 97 Lisburn Road, Belfast BT9 7BL, United Kingdom

**Keywords:** Microneedle, Scalable, Manufacture, Injection moulding, Drug delivery, Commercialisation

## Abstract

A novel manufacturing process for fabricating microneedle arrays (MN) has been designed and evaluated. The prototype is able to successfully produce 14 × 14 MN arrays and is easily capable of scale-up, enabling the transition from laboratory to industry and subsequent commercialisation. The method requires the custom design of metal MN master templates to produce silicone MN moulds using an injection moulding process. The MN arrays produced using this novel method was compared with centrifugation, the traditional method of producing aqueous hydrogel-forming MN arrays. The results proved that there was negligible difference between either methods, with each producing MN arrays with comparable quality. Both types of MN arrays can be successfully inserted in a skin simulant. In both cases the insertion depth was approximately 60% of the needle length and the height reduction after insertion was in both cases approximately 3%.

## Graphical abstract

**Figure:**
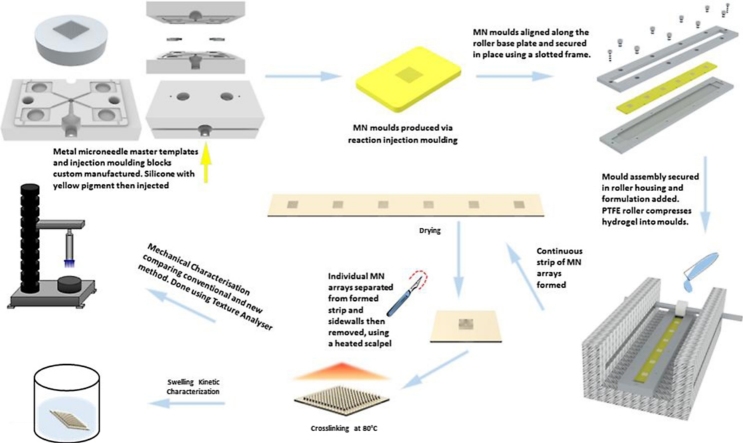

## 1 Introduction

Microneedle arrays (MN) are minimally-invasive devices that painlessly by-pass the *stratum corneum*, the principal skin barrier to topically-applied drugs, and as such are intended for drug delivery and biosensing (Donnelly et al., 2012; Singh et al., 2010a,b). They consist of a plurality of micro-projections, generally ranging from 25 to 2000 μm in height, which are attached to a base support (Donnelly et al., 2010a,b; Gittard et al., 2013). They have been extensively investigated in recent years as a means to enhance transdermal drug and vaccine delivery with a multitude of fabrication techniques, materials and geometries employed.

Different groups have investigated various types of microneedles, from in-plane (Paik et al., 2004) and out-of-plane (Donnelly et al., 2010a,b), to hollow (Gardeniers et al., 2003), solid (Ling Teo et al., 2005), macroporous (Ji et al., 2006), dissolving (Donnelly et al., 2013; Migalska et al., 2011) and swelling (Donnelly et al., 2014a; Larrañeta et al., 2015). They have been produced from a variety of materials such as glass (Martanto et al., 2006), sugar (Martin et al., 2012), metal (Martanto et al., 2004), metal coated (Zhu et al., 2012), silicon (Ji et al., 2006), solid polymer (Trautmann et al., 2005), aqueous hydrogel (Donnelly et al., 2014a) and dissolving polymers (Donnelly et al., 2013). Additionally, MN can be prepared using a wide variety of geometries, having a great impact on their performance (Gomaa et al., 2010; Olatunji et al., 2013).

As a result of the range of materials chosen and the variety of shapes designed, MN have been fabricated using a diversity of techniques, mostly from microelectromechanical systems (MEMS) technology. Fabrication techniques range from ion sputtering deposition (Tsuchiya et al., 2010), photolithography (Kochhar et al., 2013), wet and dry etching (Ji et al., 2006), photopolymerisation (Cruise et al., 1998), laser ablation and micromoulding (Aoyagi et al., 2007; Donnelly et al., 2011), layer-by-layer deposition (DeMuth et al., 2013), droplet-born air blowing (Kim et al., 2013), drawing lithography (Lee and Jung 2012) and milling (Yung et al., 2012). Yet, despite the relative degree of success in MN fabrication, there are still very few MN products on the market, in part due to the difficulty in scale-up of fabrication.

Our research group showed the ability of MN to deliver different types of molecules successfully across the skin (Donnelly et al., 2011, 2009, 2014a; Migalska et al., 2011). Recently, our work has focused on designing a MN manufacturing process capable of facile scale-up, taking account of universal acceptance criteria and GMP specifications in moving towards commercialisation (Lutton et al., 2015). A MN insertion quality control test, which could be used during manufacture, has also been developed (Larrañeta et al., 2014). In addition, research on alternative crosslinking techniques suitable for MN scale-up was conducted reducing 30-fold the crosslinking time (Larrañeta et al., 2015). Currently we produce MN arrays prepared from polymeric materials under ambient conditions in a discrete manner using an excimer laser-based method for micromoulding (Donnelly et al., 2011).

The laser machining process uses a focused optical light beam to selectively remove materials from a substrate to create a desired feature on, or internal to, the substrate. The process is non-contact, yet it has high spatial confinement. Compared to other mechanical machining techniques, laser machining, being a non-contact process, does not incur tool wear and also exhibits low heat deposition to the working piece (Brookhaven National Laboratory, 2013; Sato et al., 2014). However, laser cutting is associated with thermal effects at the cutting surface, resulting in alteration of microstructure and mechanical properties (LaserFocusWorld, 2007; Sato et al., 2014; Zaied et al., 2013). This alterations of microstructure are often associated with undesirable effects such as surface cracking, fatigue resistance and creation of microcracks in the surrounding material. In subsequent routine use of the work piece, these cracks may propagate deep into the bulk of the material and cause premature device failure (Crowson, 2006; Huang et al., 2014; LaserFocusWorld, 2007; Stavinoha, 2001; Zaied et al., 2013). Therefore, in this work we propose the use of injection moulding for the production of MN moulds. Therefore the laser process is not ideal for MN moulds production for larger scale processes.

In the present study, we describe a novel, scalable method of MN manufacture. This method is used to produce MN arrays also from polymeric materials under ambient conditions utilising a combination of injection moulding and roller casting.

## 2 Materials and methods

### 2.1 Materials

Gantrez^®^ S-97 (*M*_w_ = 1.2 × 10^6^), a copolymer obtained from the free acid of methyl vinyl ether and maleic anhydride polymers, was provided by Ashland (Tadworth, Surrey, UK). Poly(ethyleneglycol) (PEG) 10,000 Da was obtained from Sigma–Aldrich (Poole, Dorset, UK). Parafilm^®^, a flexible thermoplastic sheet (127 mm thickness) made of olefin-type material, was used as skin simulant for insertion studies and was obtained from BRAND GMBH (Wertheim, Germany). Micra-Sil^®^ antimicrobial silicone sheet was purchased from J-Flex (Nottinghamshire UK), MED-4870, MED-4830 and DDR-4320 liquid silicone rubber were all purchased from Nusil Technology (Buckinghamshire, UK), MED-4900-5 yellow dye from Polymer Systems Technology Limited (Buckinghamshire, UK), Dow Corning Silastic^®^ S RTV silicone rubber base and green curing agent from Thompson Bros. Ltd (Newcastle Upon Tyne, UK). Stainless steel and aluminium was sourced from Impact Ireland Metals Ltd. (Newtownabbey, UK) whilst poly(tetrafluoroethylene) (PTFE) was obtained from RS Components Ltd. (Northants, UK).

### 2.2 Manufacture of roller system

**Fig. 1:**
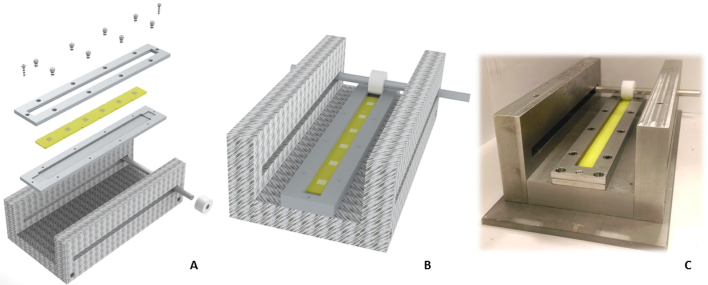
Exploded CAD image of the roller design (A) CAD image of the roller assembly (B) CAD image of the assembled roller device (C) actual assembled device.

Fig. 1 illustrates the computer-aided design (CAD) images (Solid Edge, Siemens PLC) of the designed device alongside an image of the finished device itself. Three rectangular sections, each 20 mm thick, were machined from a single sheet of stainless steel. Two sections were cut to dimensions 70 mm × 230 mm. These pieces form the lateral walls of the system (Fig. 1A). The third section was machined to 80 mm × 230 mm and formed the base (Fig. 1A). A rectangular slot of dimensions 202 mm x 8 mm was machined through both side walls in order to allow, the roller handle, an 8 mm stainless steel rod, to slide along the device. The three stainless steel sections were then bolted together to form a U-shaped housing (Fig. 1A, B and C).

A PTFE rod (14 mm thick and 23.2 mm in diameter) was used as the roller for the device (Fig. 1B). PTFE was chosen due to its hydrophobicity and anti-adherent properties. An 8 mm hole was placed through the centre for the handle to be inserted.

A roller base plate and frame, each 40 mm × 230 mm, were machined out of 5 mm thick stainless steel plates (Fig. 1A). The base plate was used to house the moulds and as such a 20 mm × 190 mm × 2 mm recess, with 3 mm radius at each corner, was machined along its centre. An additional recess of 14 mm × 12 mm × 1 mm was then machined at either end of the mould recess to act as the home position for the roller. The frame is used to secure the moulds in place during operation and to prevent leakage of the applied formulation. A rectangular section 14 mm × 214 mm was cut out of the frame plate to allow the roller to move along the housing. Eight M4 holes were drilled through both roller frame and the base plate; this was to enable the frame to be fastened to the plate. A further two M2.5 holes were drilled into each end of the roller frame, base plate and housing, to enable the assembled roller frame, moulds and base plate to be secured to the housing during operation. The eight M4 and two M2.5 holes were widened to counterbores of diameter 6 mm and 5 mm, respectively on the roller frame in order to prevent impeding the roller handle whilst in operation. Furthermore, the two additional holes at either end of the parts not only allowed the fixed placement of the mould assembly during operation but also the consistent and accurate alignment of the roller This also facilitated easy removal of the assembly post operation, ready for the next mould assembly to be rolled.

### 2.3 Manufacture of metal MN master templates

**Fig. 2:**
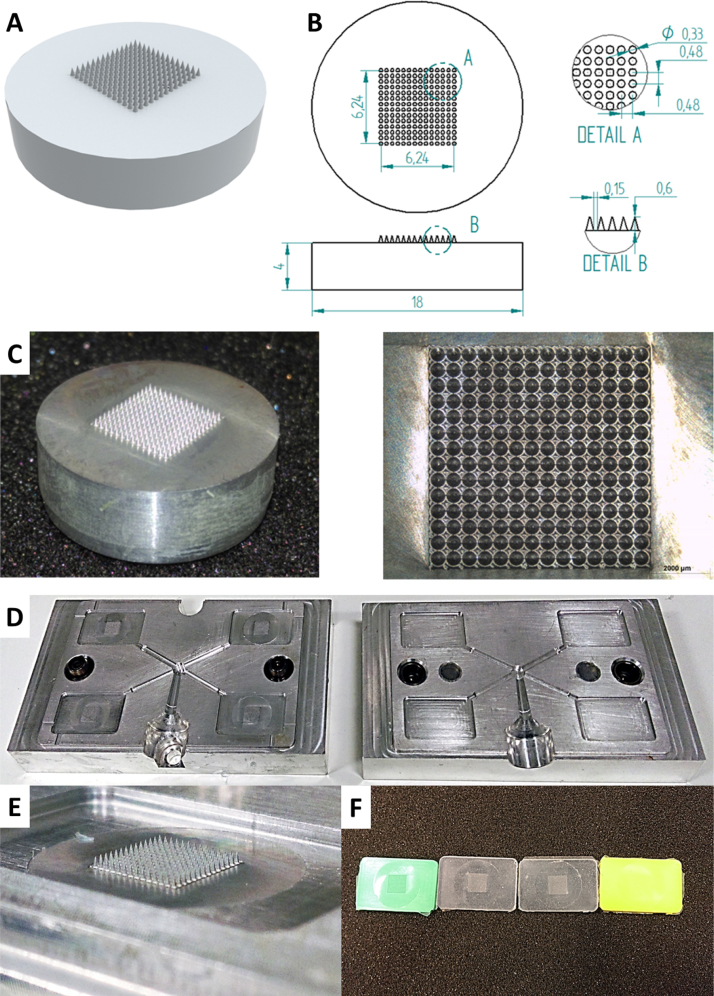
Different images of the metal MN master template: CAD design (A), draft drawing (B) and photographs (C). Photographs of the: custom made injection moulder blocks (D), metal MN master template inserted in the injection moulder block (E) and 14 × 14 MN moulds produced using different silicone grades (F). All dimensions are in millimetres.

Metal master templates of the required MNs were machined from aluminium using a 5 DMG Monoblock 60 axis mill (DMG Mori Seiki AG, Bielefeld, Germany) and the cutter used was a 0.2 mm carbide end mill. In the interest of spindle longevity, a spindle speed of 17,000 rpm and a feed rate of 30 mm per minute were chosen. The MN dimensions machined were of 14 × 14 conical microneedles, 600 μm in height, 330 μm base width and a 150 μm base interspacing producing a 480 μm pitch. Images of the CAD design, draft drawing and images of the finished metal MN master template are presented in Fig. 2A.

### 2.4 Production of silicone MN moulds

Silicone moulds were produced as described previously (Donnelly et al., 2011). However, due to the unsuitable nature of this machining process, when coupled with the roller system, an alternative method of producing moulds was designed and implemented.

Silicone elastomer MN moulds, Fig. 2F, were produced using a custom designed, laboratory-scale, injection moulding machine. Injection moulding blocks, Fig. 2D, were machined using a DMG Monoblock 60 (5 axis mill), to house the metal MN master templates Fig. 2E.

The silicone MN moulds were manufactured by mixing 200 g in total of both parts A and B of silicone elastomer Med 4870 at 1:1 wt/wt, using a DAC 600.2 Vacuum Speedmixer VAC-P (Synergy Devices, Buckinghamshire, UK). Each part was degassed separately by spinning for 2 min at 2350 rpm, then combined with 1 g of MED-4900-5 yellow dye and mixed for 1 min at 1800 rpm and then again for 20 sec at 2350 rpm. The homogenous mixture was injected into the stainless steel moulds using a SD340-30 Semco timed shot dispenser (Synergy Devices, Buckinghamshire, UK) and cured for 5 min at 140 °C.

### 2.5 Fabrication of hydrogel-forming MN arrays

#### 2.5.1 Centrifugation

In order to evaluate the quality of MN arrays produced using the novel roller system, they were compared to MN arrays produced using the previously established method, based on the use of a centrifuge and described below.

To fabricate MN using the established centrifugation method, the injection moulded (IM) silicone MN moulds were cut to the appropriate size and pasted into micromould templates as previously described (Donnelly et al., 2011). The adhesive used was uncured MED-4870 silicone, the same material used to produce the injection moulded MN moulds. Following this, aqueous blends containing Gantrez^®^S-97 (20% w/w) and PEG 10,000 (7.5% w/w) were micromoulded in the adapted silicone micromould templates, as previously described (Donnelly et al., 2011, 2010a; Garland et al., 2011; Migalska et al., 2011; Singh et al., 2010a,b, 2009). After centrifugation at 3500 rpm for 15 min, the filled moulds were dried at room temperature for 48 h, crosslinked (esterification reaction) by heating at 80 °C for 24 h and the sidewalls, formed by the moulding process, removed using a heated blade.

#### 2.5.2 Roller system

Prior to use the aqueous hydrogel was degassed either by centrifugation at 3500 rpm for 5 min or by placing the sample in a vacuum chamber. After degassing the formulation was ready to use.

**Fig. 3:**
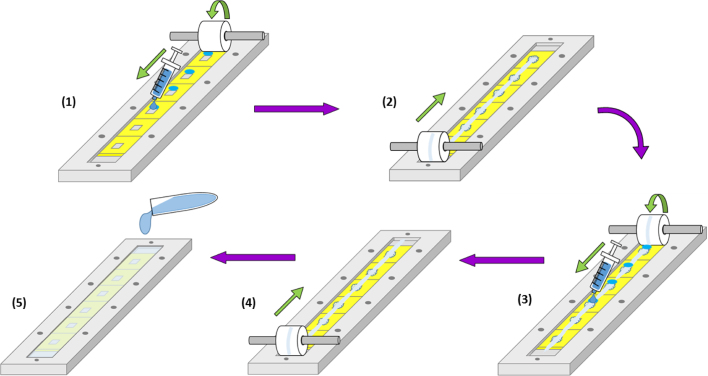
Resulting effect of using laser ablation to create MN cavities in silicone, along with the roller method.

The IM silicone moulds were aligned on the roller base plate, the roller frame bolted on top and the mould assembly then fixed to the housing. The formulation was spotted in front of the conical cavities (0.25 mL at a time) of the first four silicone moulds using a 5 mL syringe (Fig. 3.1.). Four moulds were chosen as this produced sufficient gel to dose the remaining two moulds. The roller was rolled along the entire length of the housing and then it was rolled back to the original home position (Fig. 3.2.). This process of spotting four moulds and rolling back and forth was then repeated so that the moulds experienced a compressive rolling force, four times in total (Fig. 3(3 and 4) The formulation was then layered over the exposed moulds to produce the base plate (Fig. 3(5)). The mass of hydrogel formulation may be tailored to suit the required baseplate thickness, however, in this instance 9.5 g was added to fill the frame. A schematic of this process is provided in Fig. 3. The filled mould assembly was dried at room temperature for 48 h. The roller base plate and roller frame were then separated, the moulds peeled away from the frame, exposing the fully formed MNs, and the MN strip removed using a heated blade. The strip was then cut into individual MNs and the side walls removed, also using this process, and the individual arrays were then crosslinked by heating at 80 °C for 24 h.

### 2.6 Characterisation

#### 2.6.1 Insertion test

Parafilm® M (PF) film was used as a skin simulant for MN insertion studies as described previously (Larrañeta et al., 2014). For this purpose 8 single layers of PF were combined (≈1 mm thickness), placed on a sheet of expanded poly(ethylene) for support and secured at each corner using tacks. Prior to insertion the MN height was measured using a light microscope (GXMGE-5 digital microscope, Laboratory Analysis Ltd., Devon, UK).

To perform the insertion test, MN arrays were positioned on the PF layers, needles facing down. A strip of adhesive tape was layered over the PF layers, care being taken to ensure that the tape was sticking only to the thumb tacks, with no pressure directed at the MNs. This tape ensured that the MNs were not affected by the insertion probe when it retracted. The support with MN array was then placed on the Texture Analyser testing area.

A cylindrical probe with a cuboidal end of dimensions 10 mm × 10 mm was attached to a TA.XTPlus Texture Analyser (Stable Micro Systems, Surrey, UK) in compression mode. The probe was programmed to move vertically downward at a rate of 1.19 mm/s. Once the MN array touched the support with MN array and received a trigger force of 0.49 N, the Texture Analyser began collecting data. The probe continued to move vertically downwards at the same rate until a force of 32 N had been reached; this is the maximum average force a human exerts when applying MNs (Larrañeta et al., 2014). At this point the probe stopped and remained in position, maintaining 32 N for 30 s, the time recommended for MN application (Donnelly et al., 2014b), after which time the probe retracted and the testing area was free to be cleared of the specimen.

#### 2.6.2 Optical coherence tomography

In order to ascertain the insertion depth of the MN into PF, optical coherence tomography (OCT) was used. Post insertion-test, the inserted MN array was immediately viewed using an EX1301 OCT Microscope (Michelson Diagnostics Ltd., Kent, UK). The swept- source Fourier domain OCT system has a laser centre wavelength of 1305.0 ± 15.0 nm; this facilitates real-time high-resolution imaging (7.5 mm lateral and 10.0 mm vertical resolution). The PF was scanned at a frame rate of up to 15 B-scans (2D cross-sectional scans) per second with a scan width of 5.0 mm. The 2D images were analysed using ImageJ^®^ (National Institutes of Health, Bethesda, USA). The scale of the image files was 1.0 pixel = 4.2 mm allowing the depth of MN penetration to be measured.

#### 2.6.3 Penetration and height reduction analysis using light microscopy

Once the MN arrays had been imaged using OCT, post-insertion test, the MN arrays were removed from the PF membrane. The PF layers were then unfolded and the number of holes in each layer evaluated using a Leica EZ4 D digital microscope (Leica, Wetzlar, Germany). The MN arrays were once again visualised and the heights measured and recorded. The percentage change in height was then established using Eq. (1).

(1) $$\%\text{Height}\text{ Reduction}=\frac{\text{Original}\text{ Height}-\text{New}\text{ Height}}{\text{Original}\text{ Height}}\times 100\%$$

#### 2.6.4 Compression test

A compression test determines the behaviour of materials under crushing loads. MN was compressed and deformation at various loads recorded. Normally the specimens are of uniform dimensions and regularly shaped, allowing a variety of mechanical properties to be calculated. In this instance, however, the irregular shape of the needles and the effect of baseplate leads to the test solely being used to calculate the stiffness, *S,* and the representative toughness, *AUC,* of the needles. The insertion test using Parafilm® is used to determine the feasibility of particular formulations and MN designs. It simulates the insertion of MNs into skin and therefore allows a visual guide as to the MN success in application. The compression test is used to examine the structural integrity of the needles themselves.

The MN array to be tested was attached to the end of the same probe used during the insertion test, using double-sided adhesive tape. The probe was programmed to move vertically downward, towards a metal block at a rate of 0.0167 mm/s, the rate defined for brittle materials in ISO 604 (UL LLC, 2014). When the probe received a trigger force of 0.49 N, data began to be recorded. The probe with MN array continued to move vertically downwards at the same rate until a force of 295 N has been reached; a force shy of the maximum force the texture analyser is capable of applying. At this point the probe stopped and remained in position, maintaining 295 N, for 1 s, before retracting.

Stiffness is the product of a specimen’s Young’s modulus and second moment of area (New World Encyclopedia, 2011; Ranganna, 1986; Wegst et al., 2015); it is therefore, a useful structural property indicating how a specimen of a particular shape will perform when resisting deformation. It is measured in force per unit length (N/mm) and is the gradient of a force-distance plot, such as the one recorded by the Texture Analyser. It is calculated using Eq. (2) where *Y* is the *y*-axis and *X* is the *x*-axis; the subscripts 1 and 2 are arbitrary values on each axis relating to two co-ordinates of the linear portion of the plot, with 2 being a larger value than 1.

(2) $$K=\frac{Y_{2}-Y_{1}}{X_{2}-X_{1}}$$

Representative toughness is a value denoted by AUC, the Area Under the Curve. It is a value which is an indication of toughness, i.e. the amount of energy per unit volume that a material can absorb before rupturing. This property can indicate a material’s ductility. A ductile material will absorb and dissipate much more energy than a brittle material before it fails (Keten et al., 2010; Ranganna, 1986; Zhang et al., 2006). Due to the compression test producing a force-distance plot, or curve, the AUC cannot be denoted as ‘Toughness’; toughness is the area under a stress-strain curve. However, since stress is proportional to force and strain is proportional to distance, the toughness can be inferred from the area under the force-distance curve. AUC is calculated from the integral of the force over the distance the polymer deforms before breaking, as in Eq. (3). *F* is the force corresponding to the values on the *y*-axis of the force-distance plot, *L* is the distance corresponding to the values on the *x*-axis of the force-distance plot; *L*_f_ is the distance achieved at failure. The AUC value was calculated using Prism 5 for Windows, Version 5.03:

(3) $$\int_{0}^{L_{\text{f}}}F(L)\text{d}L$$

#### 2.6.5 Fourier transform infrared spectroscopy

Attenuated total reflectance (ATR)-Fourier transform infrared (FTIR) spectroscopy was used to evaluate the crosslinking degree of Gantrez^®^/PEG polymer films and MN arrays. The IR spectra were recorded at room temperature using a FTIR Accutrac FT/IR-4100 Series (Jasco, Essex, UK) equipped with MIRacle™ software between 4000 and 400 cm^−1^ with a resolution of 4.0 cm^−1^. The obtained spectra were the result of averaging 64 scans.

The crosslinking degree of the arrays was evaluated using the area under the different carbonyl peaks, the carbonyl peak of the Gantrez^®^ acid groups (*A*_A_) ca. 1720 cm^−1^, the carbonyl peak of ester groups formed between Gantrez^®^ and PEG (*A*_E_) ca. 1770 cm^−1^ and the carbonyl peak of the anhydride peaks formed between adjacent Gantrez^®^ acid groups (*A*_AN_) ca. 1850 cm^−1^. In order to follow the crosslinking reaction a factor called Crosslinking Factor (CF) (Eq. (4)) was calculated. This factor is proportional to the crosslinking degree (Larrañeta et al., 2015).

(4) $$\text{CF}=\frac{A_{\text{E}}}{\left(A_{\text{A}}+A_{\text{E}}+A_{\text{AN}}\right)}$$

#### 2.6.6 Swelling kinetics

MN arrays (42 ± 5 mg) were weighed as *m*_o_ and then swollen in 30 mL pH 7 phosphate buffer solution (PBS) for 24 h at room temperature. At regular intervals, the films were removed, dried with filter paper to eliminate excess surface water and weighed as *m*_t_ (hydrogels). The percentage swelling, was calculated, by using Eq. (3) (Singh et al., 2009).

(5) $$\%\text{Swelling}=100\times \frac{\left(m_{\text{t}}-m_{\text{o}}\right)}{m_{\text{o}}}$$

#### 2.6.7 Statistics

All data were expressed as mean ± standard deviation. Statistical analysis was completed using ANOVA single factor tests. In all cases, *p* < 0.05 was the minimum value considered acceptable for rejection of the null hypothesis.

## 3 Results

### 3.1 Production of silicone MN moulds

**Fig. 4:**
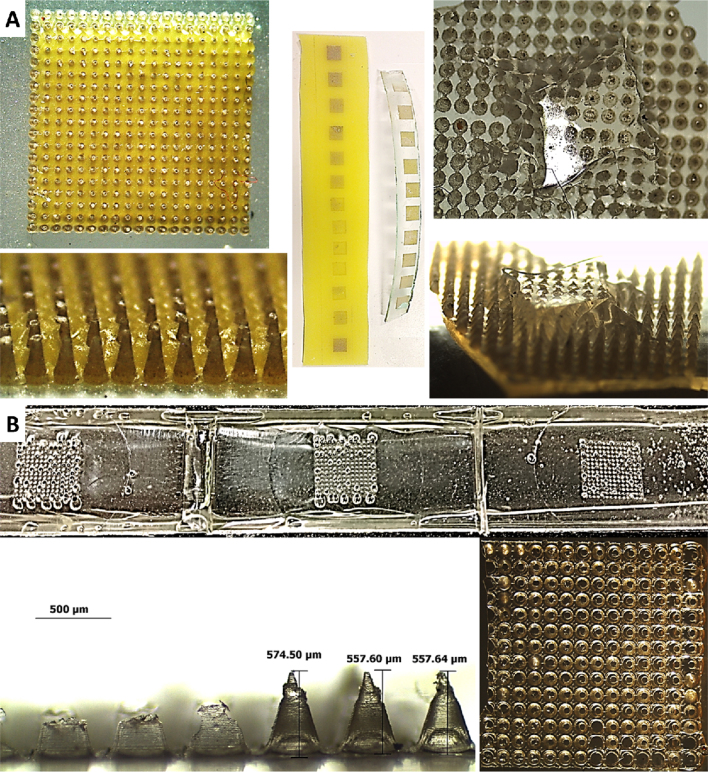
Resulting effect of using laser ablation to create MN cavities in silicone, along with the roller method (A). Images of poorly formed microneedles and excessive bubbling occurring when less suitable grades of silicone are used with the roller method (B).

As previously indicated in section 2.4, the combination of silicone MN moulds prepared by laser ablation (Donnelly et al., 2011) and the roller method of MN array production was not suitable. Fig. 4A presents images of the issues encountered when using laser fabricated silicone moulds. As can be observed, the MNs formed completely and successfully, but during the removal from the moulds, they tore the silicone. This was a similar case when different types of silicone were used. It was concluded that this issue was due to the laser process itself. As the MNs are demoulded, the weakened silicone tears, the fully formed MN arrays are removed but retain the torn silicone. This occurred with different silicone grades.

Consequently, laser engineered moulds were not a good option when combined with roller compression. Therefore, an alternative method of producing silicone was developed. Metal MN master templates and housing blocks for injection moulding were developed, as illustrated in Fig. 2D.

Nevertheless, the nature of the selected silicone strongly influenced the final MN product. Some of the selected silicones for injection moulding presented problems with MN production. The obtained MN arrays presented in some cases unformed needles and a large amount of bubbles (Fig. 4B). A number of silicone grades were trialled and the outcome was that the roller method suited higher shore hardness and tensile strength with medium elongation. Table 1 lists the silicone grades trialled and their mechanical properties. Whilst the ‘Flexsil’ antimicrobial silicone had proved successful with the roller method in forming needles, this was only available in pre-formed sheets and as such, eligible for laser ablation; therefore, MED-4870 was chosen as the silicone for MN mould production via injection moulding due to its ability to produce MN arrays without defects.

Besides, the existence of shrinkage is a point to note when using one-step injection moulding to form MN moulds. Usually the mould shrinkage of silicone rubber is 2–5%, but nonlinear shrinking can readily occur based on geometry, with difficult to predict shrinkage arising from parts of the complex shape (Corning, 2001; Hammond, 2011; Morton, 2013).

### 3.2 Fabrication of hydrogel-forming MN arrays

**Fig. 5:**
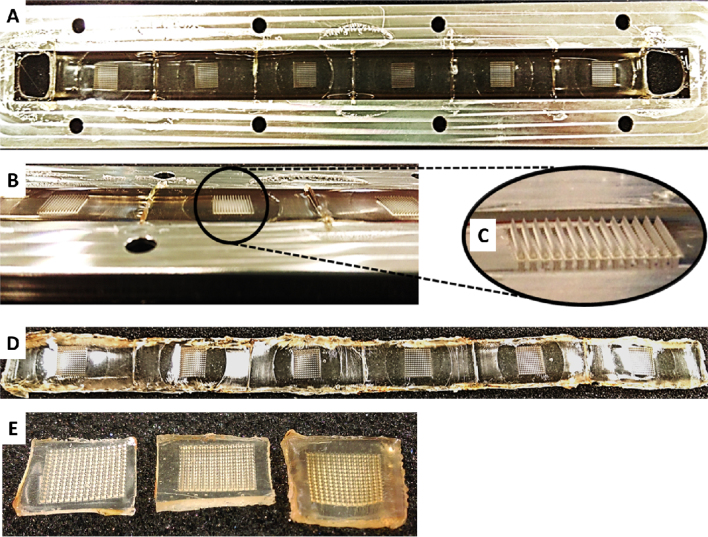
A continuous strip of MN arrays produced using the roller device, still in roller frame (A); side profile of A (B); magnified image of B (C); continuous strip of MN arrays removed from roller frame using hot scalpel (D). Comparison of (from left to right) centrifuge produced MN array and two roller produced arrays with base plate thickness increasing from left to right. (E).

**Fig 6:**
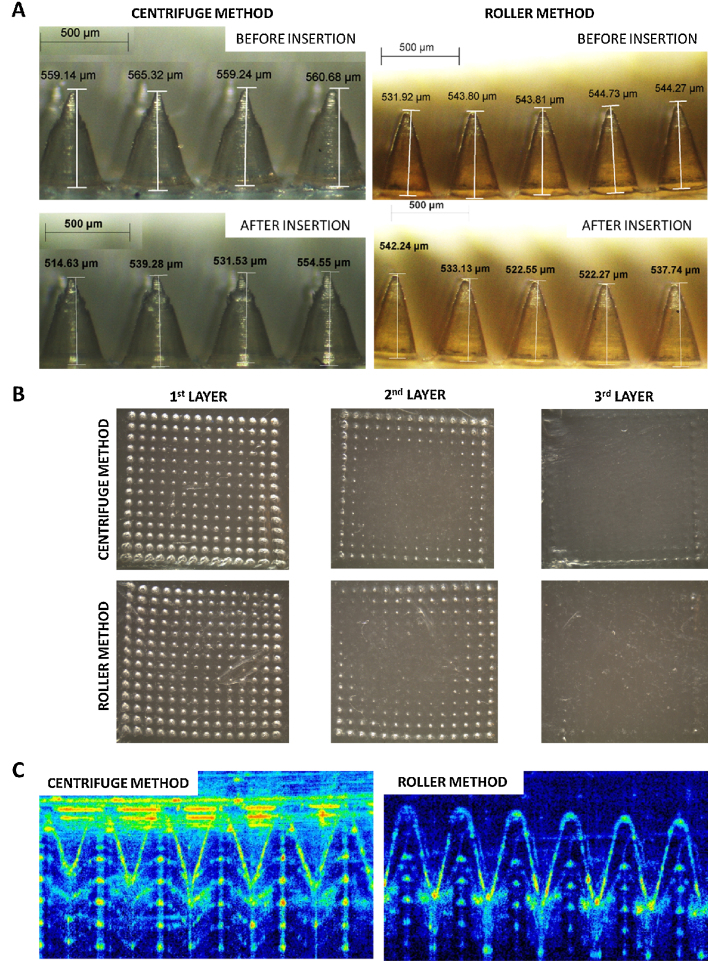
Light microscope images of MNs produced using the centrifuge method the roller method before and after insertion (A). Light microscope images of different PF layer after insertion test using centrifuge and roller produced MN arrays (B). OCT images of MN arrays produced using the centrifuge method and the roller method inserted into PF (C).

The roller device was able to successfully produce MN arrays. Fig. 5 depicts the results of the fabrication of MNs using the roller device. A comparison of a centrifuged MN array and two MN arrays produced using the roller device is presented in Figs. 5 E and 6 A (before insertion). The thicker base plate in the roller produced MN arrays is evident.

**Fig 7:**
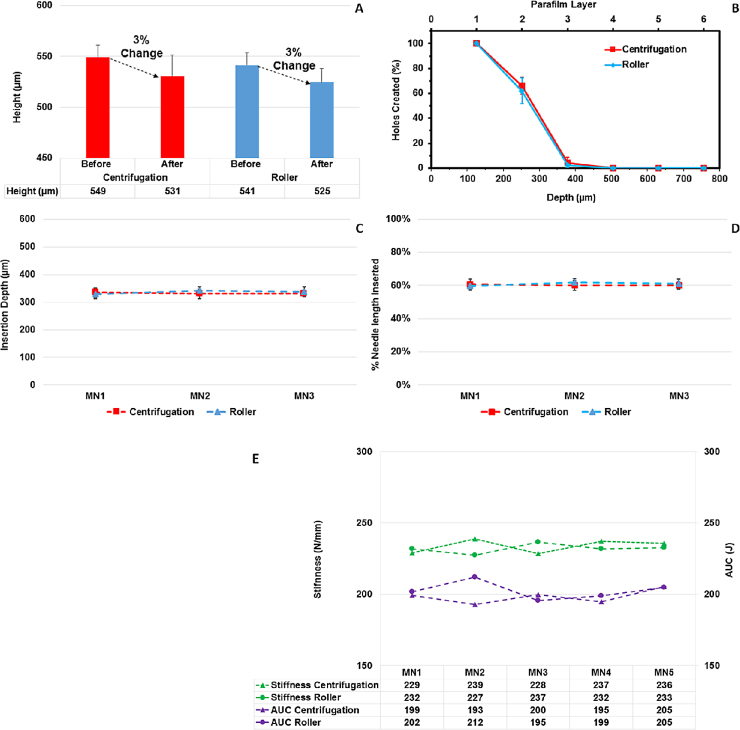
% change in height pre- and post-insertion into Parafilm^®^ for MN arrays produced using both the centrifugation and roller method (*n* = 3) (A); Comparison of the Parafilm® layers post-insertion test for MN arrays produced using both methods (B); comparison of measured insertion depth of MN arrays prepared using centrifuge and roller method measured using OCT (C) and the correlating percent of needle length inserted (D). Comparison of stiffness and AUC for MN arrays produced using both the centrifugation and roller method (E).

The average height of the roller produced MN arrays can be seen in Fig. 7A (before insertion). It can be seen that the roller method produce MN arrays slightly shorter than the ones obtained using the centrifugation method. This difference can be due to the shrinkage of moulds. It would seem that the shrinkage of moulds used in this study was ∼7%, as calculated using dimensions measured in Fig. 6A; however, due to the cavity itself being conical and a complex shape, the shrinkage is likely to be lower. Nevertheless, shrinkage occurs and should be noted for future design considerations. Nevertheless, an ANOVA single factor statistical test proved that this height difference is not statistically significant.

### 3.3 Insertion test

Images of the heights of both the centrifuge and roller produced microneedles, before and after insertion into PF, are presented in Fig. 6A. The corresponding images of the first three layers of PF used during one of each set of tests for both centrifuge and roller MN production are presented in Fig. 6B. OCT images of the MN arrays, produced using both methods, inserted into Parafilm^®^ are displayed in Fig. 6C along with the corresponding table of results in Table 2. The insertion depths obtained using both methods can be considered equivalent. Finally, the analysis of the insertion tests, including OCT, and the comparison between both methods of MN production is depicted in Fig. 7A, B and C and 7D. MN arrays produced using both methods decreased in height by 3% after insertion into PF and, as can be observed from Fig. 7B, the insertion profile for both methods is almost identical. Additionally, ANOVA single factor statistical test was completed to compare the ‘before’ heights of both methods. There was no significant difference between them. (*p* > 0.05).

### 3.4 Compression test

Results of the compression test were analysed and are graphically illustrated in Fig. 7E. The calculated stiffness and AUC for MN arrays produced using both methods are compared. As can be seen, all the stiffness and AUC obtained results for both types of MN arrays are similar. Therefore, both methods produce MN arrays that have almost identical mechanical properties and structural integrity.

### 3.5 Fourier transform infrared spectroscopy and swelling kinetics

**Fig 8:**
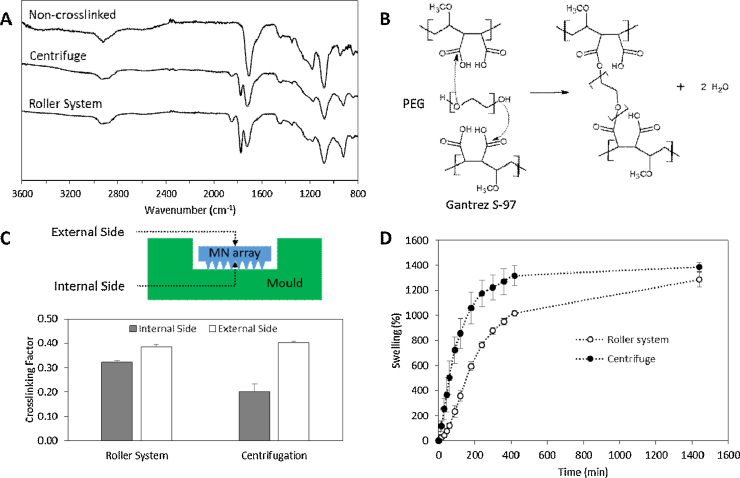
FTIR-ATR spectra of non-crosslinked Gantrez^®^/PEG films and crosslinked MN arrays prepared following the conventional (centrifuge) and the novel method (roller system) (A). Chemical reactions that take place during the crosslinking process between Gantrez^®^ and PEG (B). Crosslinking factor for crosslinked MN arrays prepared using the conventional (centrifugation) and the novel method (roller system) (Means ± SD, *n* = 3) (C). Swelling curves for crosslinked MN prepared following the conventional (centrifuge) and the novel method (roller system) (D).

Fig. 8A shows the FTIR spectra of MN arrays prepared using conventional centrifugation and roller method after the crosslinking step. As can be seen both type of MN arrays shows the characteristic ester carbonyl peak (ca. 1770 cm^−1^) formed between the Gantrez S-97 acid groups and the terminal hydroxyl groups form the PEG chains (Fig. 8B) (Larrañeta et al., 2015). This peak cannot be observed in the non crosslinked films (Fig.of formulations. As reported previously, MN prepared using the conventional centrifugation process showed different crosslinking factor depending on the side of the array (Larrañeta et al., 2015). Nevertheless, the measured CF in the internal side of MN arrays prepared using the novel proposed method are higher than those prepared using the conventional method.

Swelling kinetics of different MN arrays can be seen in Fig. 8D. Swelling curve for MN prepared using the centrifuge method shows a quicker swelling during the first hours and a slightly higher maximum swelling after 24 h than those prepared using the novel prototype (*p* = 0.08).

## 4 Discussion

As explained above, laser engineering silicone moulds were not suitable to produce MN arrays when combined with the novel roller system. This is independent of the silicone nature and, therefore, is a limitation of the laser process.

**Fig. 9:**
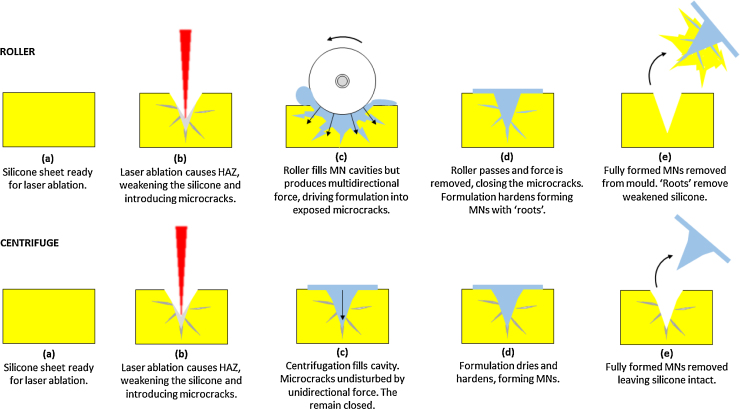
Schematic comparing the effect of using silicone moulds produced using laser ablation when fabricating MNs using the roller method and the centrifuge.

**Fig 10:**
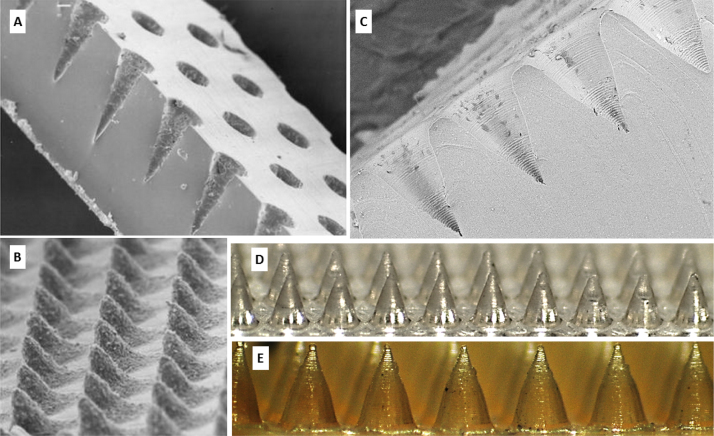
Comparison of the laser ablation method and injection moulding process of producing silicone MN moulds. Laser ablation produces a rough surface due to the production of the heat affected zone (HAZ). Injection moulding produces a much smoother surface and therefore, smoother microneedles. SEM of silicone mould produced using laser ablation method (A) and the corresponding MNs produced (B). SEM of silicone mould produced using laser ablation method (C) and the subsequent needles produced using centrifuge (D) and roller method (E).

It is thought that the low thermal conductivity of the silicone rubber reduced its ability to dissipate the laser energy sufficiently and generated a structural modified zone called heat affected zone (HAZ). This, in turn, introduced significant stress and the production of microcracks. Since the action of the roller is to compress the material, the resultant force is multidirectional, whilst with the centrifugation unit, the force is unidirectional. As the roller compresses the silicone material, the multi-directional force opens the microcracks and forces the formulation into them; as the roller passes, the multi-directional force is removed and the microcracks close, trapping the formulation. As the formulation dries, it hardens and the residual formulation in the microcracks form anchors or roots. Due to the MN cavity existing in the mechanically and structurally weakened HAZ, the adhesive bond between the hydrogel and the silicone is stronger than the cohesive bond of the silicone. As a result, when the dried strip is removed from the moulds, the silicone tears and the hardened formulation is removed intact but retains silicone. This issue was never exhibited with the centrifugation method due to the unidirectional force not exposing the microcracks and, thus, the MNs were easily removed, leaving the silicone intact. This explanation is schematically represented in Fig. 9. The act of injection moulding eliminates the issue of silicone damage, as the MN cavities are formed as part of the mould forming process; a one-step process as opposed to the two-step process of laser ablation where the silicone strip is formed first and the MN cavities subsequently formed. The result is the formation of a much smoother, defect free silicone mould. This has the added benefit of producing smoother MNs. Fig. 10 presents a comparison of moulds produced using the two-step laser ablation method and the injection moulding process, alongside images of MN arrays produced using each of the moulds.

Nevertheless not all silicone grades that were trialled were able to produce MN arrays successfully. Only silicones with specific mechanical properties were suitable for MN production using the roller method. The reason for the specific range of mechanical properties desirable for use with the roller method is unknown but thought to be due to deformation. A higher shore hardness, with a high degree of tensile strength but average elongation produces a silicone which still deforms but not to a large extent. A silicone more susceptible to greater magnitudes of deformation seemingly does not permit the formation of bubble-free, fully formed needles, perhaps as a result of increased warpage trapping more air and less inclined to retain formulation.

As the insertion tests reveal, insertion of the MN arrays into PF, mimicking insertion into skin, provides a similar result for MN arrays produced using both the centrifugation method and roller method. Each method produces MN arrays that insert to a depth of approximately 330 μm, equivalent to 60% of the total MN height, correlating with previous studies (Larrañeta et al., 2014). In addition to the insertion test, the compression test yielded similar results for the MN array stiffness and AUC.

The crosslinking step is slightly different for the novel and the conventional MN preparation process. The older process involves the crosslinking of MN arrays by placing moulds containing the arrays inside a convection oven at 80 °C for 24 h. However, in the novel process MN arrays are taken out from the moulds before the crosslinking process. Due to the thermal insulating nature of the moulds (made of silicone elastomer) the temperatures reached in the inner side of the array should be lower than the outside. Therefore, MN arrays crosslinked inside the moulds present lower CF values in the needle side than those that were crosslinked without moulds. Additionally, MN prepared using the roller system presented slightly lower crosslinking degree values in the needle side when compared to the back part of the array. This is consistent with the findings of Larrañeta et al. (2015) and may be due to the presence of residual amounts of water in the inner side. As the arrays are dried inside the moulds, the needle side of the array should present slower drying kinetics, so a small amount of water is expected to be present. This water will hinder the esterification reaction (Liu et al., 2006).

The swelling kinetics of MN prepared with the conventional and the novel process cannot be considered equivalent. The swelling process during the first hours is quicker for the MN arrays prepared using centrifugation. This difference could be due mainly to the higher crosslinking degree obtained in the needle side for the arrays prepared using the novel roller prototype. Despite this difference, the maximum swelling after 24 h for both types of MN arrays are similar.

Besides, the proposed process can be considered cost effective. No organic solvents are used and all the polymers are common excipients used in the preparation of pharmaceutical and health care products. The price of the polymers used to prepare a single MN array is around 0.09 USD. Nevertheless, this calculation was made using the prices of laboratory scale reagents and when bought in larger amounts the price will be even lower. Therefore, the cost of MN production can be lower than that of the MN produced using more expensive processes such as photolithography (Donnelly et al., 2012).

## 5 Conclusion

A novel manufacturing process for fabricating microneedles has been designed and constructed. The prototype is able to produce 14 × 14 MN arrays in a consistent way. Consequently, this novel method may facilitate scaled-up manufacture of hydrogel forming MN arrays.

The method requires silicone moulds to have cavities with a smooth surface. As a result, custom made metal MN master templates and corresponding injection moulding blocks to house the templates were designed to allow injection moulding of the moulds. Silicone of high shore hardness and tensile strength but medium elongation is an ideal grade for use with the roller device.

The MN arrays produced using this method have been characterised and compared with the traditional centrifugation method. The results prove that the roller method produces MN arrays that are of comparable quality and it is therefore an acceptable method of MN production. Additionally, the described prototype could be applied to a wide variety of formulations for MN moulding.

## Figures and Tables

**Table 1:** List of silicone grades trialled with the roller method.

	Shore Hardness (A)	Tensile strength (MPa)	Elongation%	Tear strength N/mm
Dow Corning Silastic S	26	6.9	900	24.5
DDR-4320	25	3.4	300	–
Flexsil Antimicrobial^a^	62	10.5	360	14.7
Med 4870^a^	70	10.3	415	40.6
Med 6019	75	9.0	65	N/A

a Denotes silicone grades, which produce successful, fully formed MN arrays.

**Table 2:** PF insertion depths of MN arrays produced using the centrifuge method and the roller method measured using OCT.

	Centrifuge	Roller
Average insertion depth	332	336
Average% insertion	60%	61%
SD	3%	2%
